# Sentiment analysis of COVID-19 tweets from selected hashtags in Nigeria using VADER and Text Blob analyser

**DOI:** 10.1186/s43067-023-00070-9

**Published:** 2023-01-16

**Authors:** Odeyinka Abiola, Adebayo Abayomi-Alli, Oluwasefunmi Arogundade Tale, Sanjay Misra, Olusola Abayomi-Alli

**Affiliations:** 1 Department of Computer Science, Federal University of Agriculture, Abeokuta, Nigeria; 2 Department of Computer Science and Communication, Østfold University College, Halden, Norway; 3 Department of Software Engineering, Kaunas University of Technology, Kaunas, Lithuania

**Keywords:** Sentiment analysis, Nigeria, COVID-19 tweets, Hashtags, VADER, TextBlob analyser

## Abstract

**Background:**

Information is essential for growth; without it, little can be accomplished. Data gathering has seen significant changes throughout the previous few centuries because of the certain transitory medium. The look and style of information transference are affected by the employment of new and emerging technologies, some of which are efficient, others are reliable, and many more are quick and effective, but a few were disappointing for various reasons.

**Aims:**

This study aims at using TextBlob and VADER analyser with historical tweets, to analyse emotional responses to the coronavirus pandemic (COVID-19). It shows us how much of a sociological, environmental, and economic impact it has in Nigeria, among other things. This study would be a tremendous step forward for students, researchers, and scholars who want to advance in fields like data science, machine learning, and deep learning.

**Methodology:**

The hashtag ‘COVID-19' was used to collect 1,048,575 tweets from Twitter. The tweets were pre-processed with a Twitter tokenizer, while TextBlob and Valence Aware Dictionary for Sentiment Reasoning (VADER) were used for text mining and sentiment analysis, respectively. Topic modelling was done with Latent Dirichlet Allocation and visualized with Multidimensional scaling.

**Results:**

The result of the VADER sentiment returned 39.8%, 31.3%, and 28.9%, positive, neutral, and negative sentiment, respectively, while the result of the TextBlob sentiment returned 46.0%, 36.7%, and 17.3%, neutral, positive, and negative sentiment, respectively.

**Conclusion:**

With all of this, information from social media may be used to help organizations, governments, and nations around the world make smart and effective decisions about how to restrict and limit the negative effects of COVID-19. Also, know the opinion and challenges of people, then deal with the problem of misinformation. It is concluded that with popular belief a significant number of the populace regards COVID-19 as a virus that has come to stay, some believe it will eventually be conquered.

## Introduction

Coronavirus known as COVID-19 is a virus that broke out globally in the year 2019 from Wuhan, China, and for a long time was the most widely disseminated disease and most widely discussed in the world. COVID-19 affected many sectors of the world, ranging from health, the economy, and education just to mention a few. As at the time of this study, over 119 million individuals have been infected by the disease, 67.2 million have recovered, and more than 2.63 million were recorded dead around the world [1]. This showed it is perhaps the most natural infection outbreak in the most recent twenty years in the century [2]. With statistics obtained from social media usage, especially Facebook and Twitter, the advent of technology has allowed us to comprehend the impact of this pandemic in numerous sectors. It is well known that information travels quickly over the Internet, resulting in a wide range of emotions among social media users, particularly on microblogs like Twitter. Within the twentieth century, social connection advanced to a technical level, allowing people to connect with others all over the world to promote society's acceptance. Twitter is one of the social programmes that is widely used for opinion polling, with 100 million users posting 250 million tweets [3]. Infection with COVID-19 became a threat not only to public health but also to global development. COVID-19 could be a highly contagious disease that targets the respiratory system and the lungs. According to studies, COVID-19 is coronavirus-related and shares characteristics with an ailment that first surfaced in 2003 under the name of severe acute respiratory syndrome (SARS). Beginning in China, the SARS virus infected 29 countries.

With sentiment analysis, a relationship can be established between the content of a tweet and the emotions of the composer. Hence, mining opinion on social media microblogs presents opportunities to extract meaningful insight from the public, especially on trending issues [4]. Twitter is a social platform in which users can express their thoughts in less than 280 characters per message on different ranges of topics. By applying sentiment analysis techniques to tweets, important bits of information are gotten with respect to open opinions on the pandemic, open health measures, and the mental effect on living all through the period of the pandemic [5]. As a result of a succession of disease management measures such as travel barriers, community isolation, and social distance, the infectious coronavirus has made the public reliant on online data for individuals to stay educated and engaged. Individuals expressed their fears of contamination and shock with respect to contagious diseases on social media, in addition to their sentiments around contamination control methodologies. They also showed emotional responses to a few health-unrelated topics, such as the economy and the worldwide natural effect of COVID-19 widespread, according to studies. Meanwhile, racist speculations and hostile statements about the populace, which have been shown to provoke negative attitudes, have grown in popularity on social media. Anxiety and despair have increased because of increased social media exposure [6].

As indicated in Abdulaziz et al. [7], an analysis of Twitter data, particularly individual’s emotions, is valuable in numerous areas like the financial exchange, managing disasters, voting in elections, and preventing crime. The traditional method of sentiment analysis is a long and tedious process and oftentimes, certain feedback is not seen. With a large amount of user-generating content on social media, it is difficult to read, analyse and interpret all social media reviews because data generated online are usually disorganized and very unstructured. Drawing insights from online sources is a challenging task, and the quality of opinions cannot be guaranteed since people post their content freely [8]. Twitter is one of the important sources that can be used to solve this problem by performing sentiment analysis on the data extracted online. This allows researchers to gain insights into users’ opinions towards the coronavirus pandemic in real time despite the challenges posed by data volume and structure [9].

This study aims to underscore the sentiments and opinion of Twitter users in Nigeria towards the COVID-19 disease by collecting a corpus of COVID-19 text from historical tweets, pre-process the text, conduct sentiment analysis and topic modelling and then evaluate the performance of the developed model. The remaining part of the paper is as follows: Sect. "Review of related work" discusses the related work, Sect. "Methodology" provides a description of the methodology for the study, Sect. "Implementation and results" presents the implementation and discussion of the results obtained, while the paper concludes in Sect. "Conclusion".

## Review of related work

This section presents the literature reviews on the study.

The five tops subjects attached to the COVID-19 pandemic were: economy and trade, health care, emotional support, psychological stress, and social change. These topics conveyed the biggest worries for the populace [10].

Gao et al. [11] explored the outcomes regarding mental prosperity by the individuals who were constantly revealed to online media during this pandemic. It was stated that large numbers of mental health concerns, such as anxiety or bitterness, had a good association with the widespread use of social media during the COVID-19. In Konac et al. [12], usage of social media as a source of information was associated with extending conspiracies concerning the pandemic, along with propagation of several unverified health protection practices.

The severe situation in which people are unable to leave their homes necessitates an investigation into what people are thinking about during the pandemic [13]. Overall sentiments conveyed in tweets during the pandemic were more positive, meaning that the public remained hopeful even while confronting a global public health issue.

The highest degree of positive sentiments suggests that a lot of people were carefree about the seriousness of COVID-19 at the early stage of the pandemic. One important note is that tweets created in states experiencing lower rates of infection were generally positive, while those states more directly influenced by the pandemic were negative. The negative sentiment catchphrases recommend that tweets might be an approach to express negative feelings around the consequences of COVID-19 constraints. Negative expressions normally included "know" and "think", words that identify with information and information sharing [10].

A summary of related work on COVID-19 analysis using Twitter data is provided in Table 1.

**Table 1:** Summary of related work on COVID-19 analysis using Twitter Data

S/n	Author	Title	Methodology	Dataset	Results	Limitation
1	Samuel et al. [14]	COVID-19 Public Sentiment Insights and Machine Learning for Tweets Classification	Descriptive textual analytics Positive sentiments were assigned 1, negative sentiment was assigned 0	Public reliance on social media Tweets	The study explores the viability of machine learning classification methods	There was sufficient directional support for Naïve Bayes and Logistic classification for short to medium length tweets
2	Bania [15]	COVID-19 Public Tweets Sentiment Analysis using TF-IDF and Inductive Learning Models	Textual analytic, natural language processing (NLP) and use of artificial intelligence (AI) techniques	40,000 tweets collected manually from twitter site between 3/07/2020 and 11/07/2020	Experimental results suggest that Random Forest and Bernoulli’s Naïve Bayes models performed better than the other two classifier models	Twitter data alone may not be sufficient to reflect the general mass sentiments for a nation or for the states
3	Shorten et al. [16]	Deep Learning applications for COVID-19	language modelling: Natural Language Processing, Computer Vision, Life Sciences, and Epidemiology	Nil	Result is based under the true positive, false positive rate curve (AUC), and precision-recall	Interpretability, Generalization Metrics, Learning from Limited Labelled Data, and Data Privacy
4	Alanezi and Hewahi [17]	Tweets Sentiment Analysis During COVID-19 Pandemic	k-means clustering and MiniBatch k-means clustering using COVID-19 tweets	WHO data are generated using R Language and have 3500 tweets. Bahrain ministry of health dataset is collected using R Language and has 3500 tweets. The English tweets dataset is having 34 attributes and 23,490 instances. The Arabic tweets dataset is having 34 attributes and 13,088 instances	The highest number of words are classified as neutral with 7184 as 43.2%, then positive words with 4870 as 29.3%. The negative words with 4572 as 27.5%	There are a lot of spam tweets in Arabic with commercial tweets as advertisements that affect the quality of the data and take more time in cleaning
5	Ramírez-Sáyago [18]	Sentiment analysis from Twitter data Regarding the COVID-19 Pandemic	NLP tasks and developing classifiers based on algorithms such as Naïve Bayes and Decision Trees	78,000 tweets that contain selected phrases	Fear is the most common emotion, followed by surprise, sadness, happiness, and anger	Validation of API request by Twitter and the delay in accepting the request by Twitter
6	Petersen and Gerken [19]	COVID-19: An exploratory investigation of hashtag usage on Twitter	The data were analysed using data science and natural language processing libraries. Qualitative analysis was performed using thematic analysis	A total of 28.5 M tweets have been retrieved, of which 6.9 M tweets included hashtags	The top three themes regarding the number of hashtags used were related to COVID-19, identifying information, interventions, and geographical tagging	907 k different hashtags were used. Of these, only 1192 hashtags were used more than 1000 times. The qualitative analysis resulted in 13 themes
7	Shi et al. [20]	Social Bots’ Sentiment Engagement in Health Emergencies: A Topic-Based Analysis of the COVID-19 Pandemic Discussions on Twitter	The samples were analysed with Linguistic Inquiry and Word Count (or LIWC) This automated text analysis application extracts structural and psychological observations from text records	This study used a self-designed Python script to collect tweets and authors	A total of 118,720 unique users contributed to these tweets, of which 10,865 (9.15%) were social bots, 107,478 (90.53%) were humans, and 375 (0.32%) were unknown users	It is challenging for researchers to demonstrate the political, social, or economic motivation of social bots. we cannot conclude the intention of social bots based on their sentiment expressions
8	Dubey [21]	Twitter Sentiment Analysis during COVID-19 Pandemic	The data are being analysed using “RStudio” and are presented with the help of a cloud, graphs and tables	The data are taken from Twitter (#Covid19#Coronavirus) and total of 10,000 tweets are into consideration to find out the emotion, state and sentiments of people	The top emotion which the people are showing is positive as the score is 62 and are hoping that everything would get well soon and they will return to normalcy which is a good sign for the world	Nil
9	Chakrabortyet al. [22]	Sentiment Analysis of COVID-19 tweets by Deep Learning Classifiers- A study to show how popularity is affecting accuracy in social media	The claims have been validated through a proposed model using deep learning classifiers with admissible accuracy up to 81% and fuzzy logic for taming the fuzziness of sentiments	a dataset containing 226,668 tweets collected within a time frame and have been analysed which contrastingly show that there were a maximum number of positive and neutral tweets tweeted by netizens	The validation of this research was proposed using deep learning classifiers with an accuracy of 81%	The research demonstrates that though people have tweeted mostly positive regarding COVID-19, yet citizens were busy engrossed in re-tweeting the negative tweets and that no useful words could be found in WordCloud or computations using word frequency in tweets
10	Abdulaziz et al. [7]	Topic-based Sentiment Analysis for COVID-19 tweets	The Latent Dirichlet Allocation (LDA) was adopted for topics extraction, whereas a lexicon-based approach was adopted for sentiment analysis	There were 636,798,623 tweets dataset which was decreased to approximately 600,000 tweets	The findings showed 91% accuracy of tweets with Naïve Bayes while 74% with the logistic regression classification	The dataset contains tweet-ID only; therefore, it got more time to rehydrate it and then extract all information of tweets using the code

## Methodology

This section presents the methodology for the study including data collection, pre-processing and analysis.

### Method of data collection

Historical COVID-19 tweets were collected using the Scweet Twitter Library. Popular hashtags connected to the coronavirus in Nigeria were used as keys. The tweet content and other metadata such as timestamp, location, language, and number of retweets were stored. Only tweets in English were collected.

**Fig. 1:**
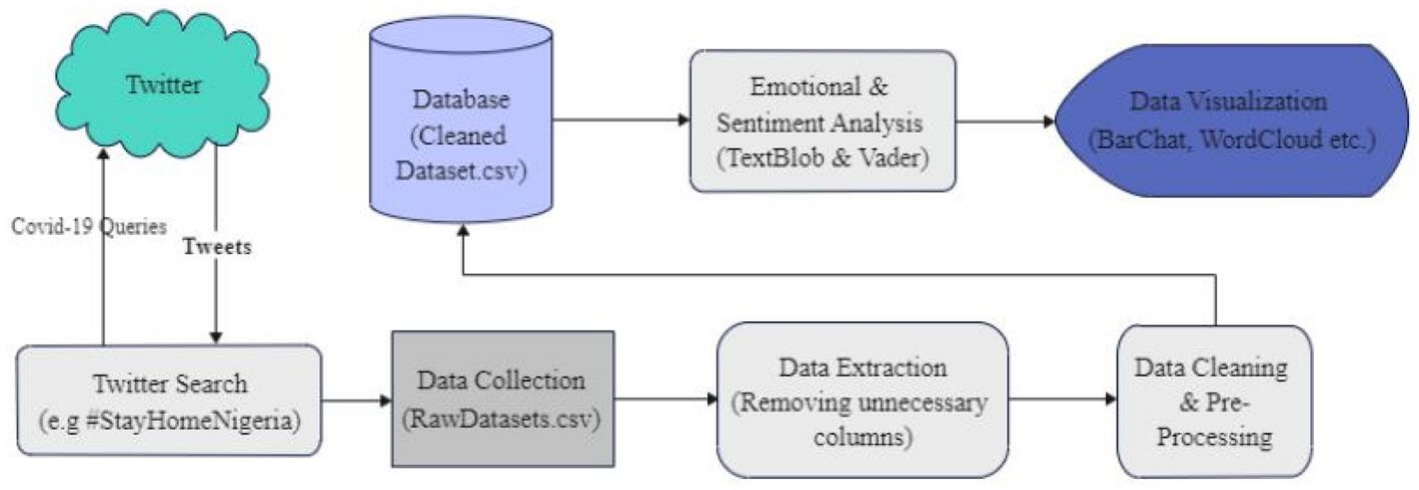
The architecture of the sentiment analysis of COVID-19 tweets in Nigeria

Figure 1 depicts the architecture of the COVID-19 sentiment analyser showing the study approach as divided into different categories which are: data collection, pre-processing, analysis, and visualization.

The architecture's modules are described in the following sections.

#### Data collection

In this study, Twitter data were used due to the level of its popularity and that its content is typically smaller in both actual and aggregated file size for user’s sentiment analysis and reaction to the coronavirus pandemic [7].

#### Data pre-processing

During this step, the obtained data are processed to prepare it for the following stage. Several stages are included in this stage:

1. *Punctuation Removal* The purpose of the punctuation removal technique is to eliminate punctuation marks from text data. This is yet another text standardization method that will allow 'hurray' and 'hurray!' to be treated in the same way. Depending on the use case, the list of punctuations to exclude must also be carefully chosen. The string. punctuation in Python, for example, contains the following punctuation symbols. (!"#$%&\'()*+,−./:;<=>?@[\\]^_{|}~`). More punctuations can be added or removed depending on the need.
2. *Remove Numbers* Remove any numbers that aren't related to the research. Regular expressions are commonly used to eliminate numerals.
3. *Remove duplicates/frequent words* Stopwords are eliminated based on language data. However, if a domain-specific corpus exists, this phase will remove any frequent terms that aren't important.
4. *Remove URLs* The next stage in the preparation process is to remove any URLs from the data. When conducting a Twitter analysis, for example, there is a good probability that the tweet will contain a URL. It will almost certainly be necessary to remove them to do future research.
5. *Remove Whitespaces* A text may contain extra whitespace which is not desired as they increase the text size and do not add any value to the data. Hence, removing extra whitespace is a trivial but important text pre-processing step.
6. *Lowering the text* The lower casing is a text pre-processing technique that is widely used. The purpose is to transform the input text to the same case as the output text, so that 'text’, 'Text', and 'TEXT' are all treated the same. This is especially useful for text featurization techniques like frequency, as it helps to combine similar terms, reducing duplication and ensuring accurate counts.
7. *Stop words removal* Stop words are frequently used words that have been eliminated from the text since they provide no value to the study. These concepts are either meaningless or have no significance at all. A list of terms that are stop words in English may be found in the NLTK library. Some of these words are Me, myself, we, our, ours, ourselves, you're, you've, you'll, some, you'd, your, my, yours, yourselves, he, most, other, such, no, nor, not, you, only, own, same, so, then, too, I, very, s, yourself, t, can, will, just, don't, should, should've, now. However, using the provided list as stop words is not required because they should be picked carefully based on the study.
8. *Lemmatization* It keeps the sense of the term while stemming it. Lemmatization makes use of a pre-defined dictionary to keep word context and check the word in the dictionary as it gets smaller.
9. *Tokenization* This is the process of breaking down the text into individual tokens. Converting a corpus of text into tokens of phrases, words, or even characters is possible. This research usually aids in the conversion of text into word tokens during pre-processing, as they are required for many NLP procedures.

#### Data analysis

At this point, all data should be examined and recognized based on the primary goal of the study, such as polarity identification, sentiment analysis, or frequency analysis.

#### Visualization of data

Data visualization is the process of presenting complicated information using simple diagrams and charts. Data visualization can present data-driven tales while also allowing users to see data trends and linkages.

1. *DataFrame* Pandas DataFrame is a two-dimensional size-mutable tabular data format with labelled axes that can be heterogeneous (rows and columns). A data frame is a two-dimensional data structure that organizes data in a tabular format in rows and columns.
2. *Bar Chart* They are very useful for data visualizations and the interpretation of meaningful information from datasets. One of the libraries used to create a bar chart is matplotlib, which is a maths library widely used for data exploration and visualization.
3. *Seaborn* Seaborn is also a visualization library based on matplotlib and is widely used for presenting data. The library can be imported as sns.
4. *pyLDAvis* Learning about how subjects relate to one another, including potential higher-level structure between groups of topics, is aided by showing the information included in a topic model using an intertropical distance map.

### Research method

All data were processed and then identified based on the study's principal purpose, such as polarity identification, sentiment analysis, or frequency analysis, at this stage. The data studied were based on the content of the tweet, as well as some other data.

#### Natural language processing

The process of developing software and services that can understand human languages is known as natural language processing (NLP). Speech recognition, for example, in Google Voice Search, comprehending what the content is about, and sentiment analysis is all instances of NLP in action. Running through some of the basic procedures with the Natural Language Toolkit is the best approach to demonstrating NLP (NLTK).

The most widely used natural language processing software is NLTK, which was created in Python to cope with human language data. It comprises classification, tokenization, and lemmatization text processing packages, as well as user-friendly interfaces like WordNet.

#### Sentiment analysis

Sentiment analysis is a step-by-step method of analysing textual data using natural language processing algorithms. Hidden information could be discovered using VADER and TextBlob Sentiment Analysis. This information is usually hidden in collected and stored data. The analysis can show how positive or negative the text data is. There are many practical applications for this process. For example, this report could help companies in creating customer-oriented strategies. With the enhancement in artificial intelligence algorithms, it is much easier now to handle and study textual data. Moreover, these algorithms are getting high accuracy rates for their assumption of sentiments related to data. Another major example of using sentiment analysis is in Social Media channels. Platforms like Facebook and Twitter are using this technique for preventing the spread of fake and hateful news.

In Python, there are numerous packages that perform sentiment analysis using various methods. The following are some of the most popular approaches and packages that will be employed in this paper:

##### Sentiment analysis with TextBlob

TextBlob is a text processing package for Python 2 and 3. It provides a basic API for doing common natural language processing (NLP) tasks such as part-of-speech tagging, noun phrase extraction, sentiment analysis, classification, and translation. For a given input sentence, it additionally returns two properties:

1. *Polarity* The polarity determines the polarity of the emotions represented in the statement under consideration. It has a range of [− 1,1], where − 1 indicates negative sentiment, and + 1 indicates good sentiment.
2. *Subjectivity* Subjectivity is used to determine the speaker's personal states, such as emotions, beliefs, and opinions. It has a range of [4], with a number closer to 0 indicating that the sentence is objective and is founded on facts, and vice versa.

TextBlob will ignore terms with which it is unfamiliar, and it will consider words and expressions to which it can apply extremes and midpoints to arrive at the final score, which is defined as;


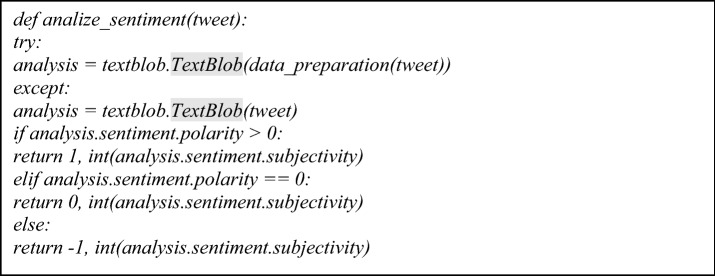


##### Valence-aware dictionary and sentiment reasoner (VADER)

It calculates text sentiment using a collection of lexical features (for example, words) that are categorized as positive or negative based on their semantic orientation. VADER sentiment gives the likelihood of a given input statement being positive (+ 1), negative (− 1), or neutral (0), as well as points. It can be optimized for social media data and generate good results when used with data from Twitter, Facebook, and other social media networks. Its outcome demonstrates the polarity of the word and their probabilities of being pos, neg neu, or compound, as defined as;


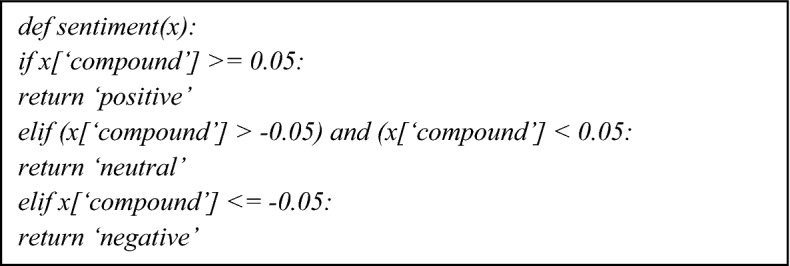


*Calculate Compound Score* VADER searches the text for known sentimental features, modifies the intensity and polarity according to the rules, adds the scores of features identified within the text, and then normalizes the final score to (− 1, 1) using the function:

$$\frac{x}{{\sqrt {x^{2} } + \alpha }}$$

The value of alpha in VADER is 15, which is close to the maximum predicted value of *x*. VADER returns the percentage of positive, negative, and neutral sentiment elements in addition to the sentence's compound score. Table 2 shows the distribution of sentiment scores of TextBlob and VADER.

**Table 2:** Table showing the sentiment score algorithm

Sentiment results	VADER score	TextBlob score
Positive	≥ 0.05	> 0
Neutral	> − 0.05 and < 0.05	= = 0
Negative	≤− 0.05	< 0

### Topic modelling

Topic modelling is a statistical technique for determining the abstract "themes" that appear in a collection of texts. Topic modelling is a text-mining technique for discovering latent semantic patterns in the body of a document. Given that a document is about a specific topic, certain words should be expected to appear more or less frequently: "dog" and "bone" should appear more frequently in documents about dogs, "cat" and "meow" should appear more frequently in documents about cats, and "the" and should appear roughly equally in both [23].

### Method of topic modelling

The Latent Dirichlet Allocation (LDA) method of topic modelling was used in this study. LDA is a widely used text mining method that classifies text in a document into one of several topics using statistical (Bayesian) topic models. It generates a topic per document and word per topic model based on the Dirichlet distribution. The LDA model is a generative model that tries to recreate the writing process. As a result, it attempts to construct a document based on the given topic.

## Implementation and results

This section is based on the process used to implement sentiment analysis and topic modelling based on COVID-19 historical tweets. The goal of this implementation is not only to perform exploratory data analysis (EDA) instead to know the populace’s opinion about the current pandemic across the world, particularly in Nigeria, which is the focus of this paper, and to see the frequency of how each topic had been discussed using topic modelling.

### Mining COVID-19 text from online tweets

**Fig. 2:**
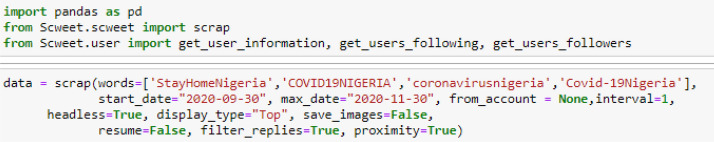
Searching of tweets using python library by keywords or word list

As shown in Fig. 2 at the first attempt, the total number of tweets mined was 11,859 starting November 2019 to May 2021. UserName, Text, TimeStamp, Likes, Retweets, Embedded_text, Comments, Image link, Tweet URL, and UserScreenName were all included in the Twitter search. After which some additional tweets of 1,036,716 were gotten from a secondary source. With the challenge surrounding Twitter API the limitation in the time frame of tweeting the needed data, a python library “*Scweet*” was adopted, this allows the streaming of data up to the preferred date and can generate a large sum of data.

**Fig. 3:**
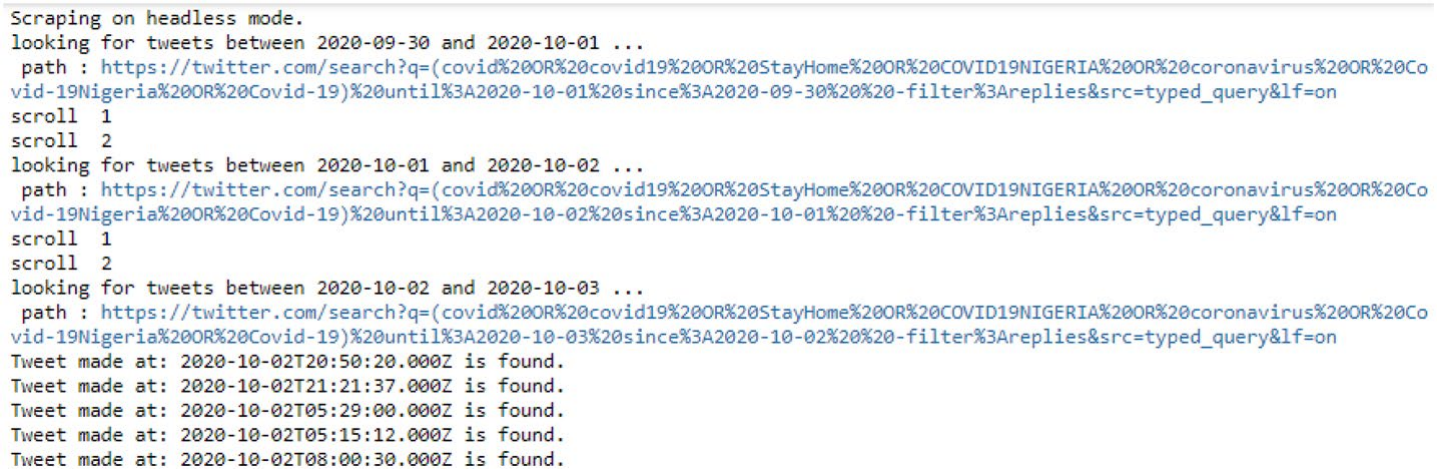
A progress showing if Tweets are available in the selected time frame

The tweet query searched was the hashtags #StayHomeNigeria, #covid19nigeria, #coronavirusnigeria, #COVID-19Nigeria, #NCDC, #FMOH. As shown in Fig. 3, the python library has no limitation on tweets, and it only returns the available results. The connection could be lost during the streaming of data, which is the more reason why the streaming was done bi-monthly on several attempts to change the date when each process finishes. After all, the process had been completed, and the data gotten were merged and exported as a single Comma Separated Value (.csv) format or Excel (.xlsx) format.

### Pre-processing of the COVID-19 tweets datasets

For the dataset to be suitable for the analysis, there was need for some cleaning and transformations generally referred to as pre-processing. This involes tasks such as conversion of all the text to lower case and dropping of irrelevant columns as described below:

#### Removal of duplicate and unwanted tweets

The corpus contained 1,048,575 tweets originally but 115,914 was removed as retweets, duplicates and irrelevant tweets containing words like “i follow”, “ifb”, “instant follow”, “cases rise”, “follow back”, “fb asap”, “lets follow”, “following follows”, “im following”, “live scores”, etc. Finallly, we have 932,661 unique valid tweets.

#### Remove hyperlinks, Twitter marks, and styles

After the removal of duplicate and unwanted tweets, some commonly substrings like the hashtag, handles, retweet marks, multiple spaces, punctuation, special characters, and hyperlinks were removed using REGEXP library. The search pattern was defined using the sub () method to remove matches by substituting with an empty character (i.e. ‘ ‘) since the focus on the text.

#### Remove empty values

After removing unwanted tweets, it was discovered that some tweets have no content which account to 100,000 “NAN” values, this resulted to further data cleaning by removing the empty values, Thus, leaving only 832,661 tweets using a “*dropna”* function.

#### Word cloud

**Fig. 4:**
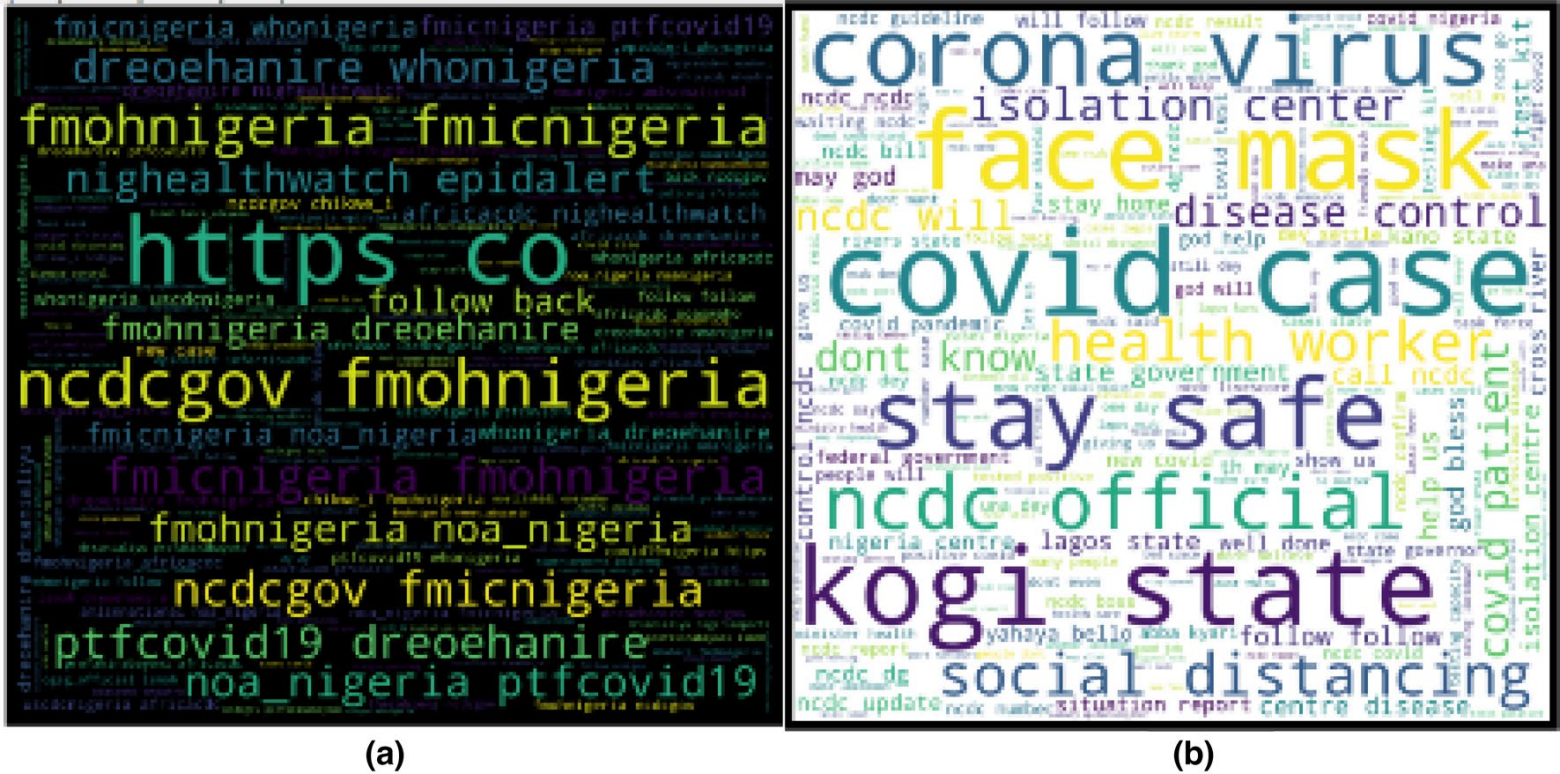
Word cloud representation of tokens in the dataset **a** raw dataset and **b** cleaned dataset

The word cloud is a graphical representation of the spread and frequency of unique tokens in the dataset. Text with higher frequency in the corpus is larger, while the less frequent ones are smaller in size. Figure 4 shows the word cloud of tokens in the initial raw dataset and the pre-processed dataset.

### Conducting sentiment analysis

For sentiment analysis of the COVID-19 tweets, two sentiment analysis approaches were employed, namely TextBlob and VADER analyser using 832,661 unique tweets.

#### TextBlob sentiment analysis

**Fig. 5:**
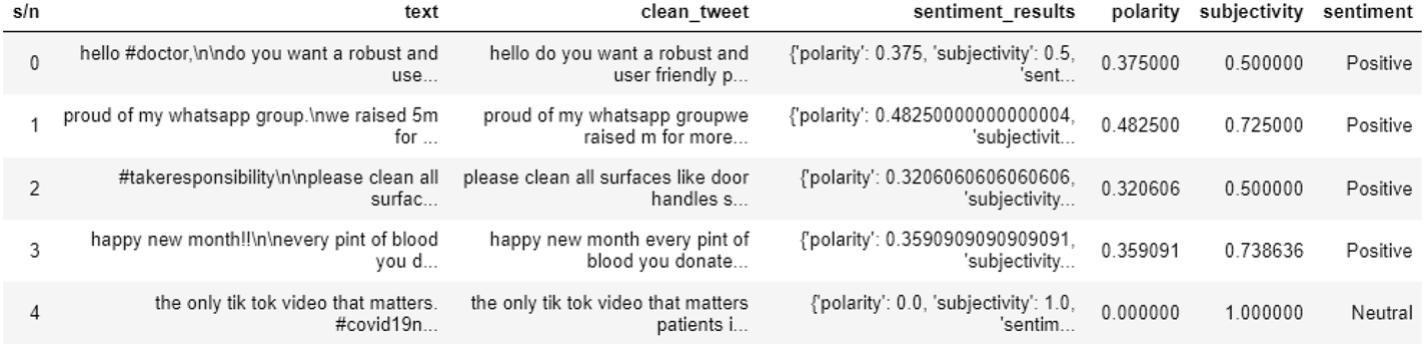
Diagram showing category distribution of sentimental analysis using TextBlob

As shown in Fig. 5, this analysis classifies the text into two forms of sentiment which are polarity and subjectivity, and these sentiment results were shown, respectively.

The result of the study shows negative, positive, and neutral sentiments. 832,661 instances were used, 305,345 of the instances prove that the authors are positively sentimental. 383,136 are neutral showing they are not sentimental in their opinions, while 144,180 are negatively sentimental as shown in Table 3.

**Table 3:** Diagram showing the frequency distribution of sentimental analysis using TextBlob

Sentiment	Count	%
Neutral	383,136	46.0
Positive	305,345	36.7
Negative	144,180	17.3

#### VADER sentiment analysis

**Fig. 6:**
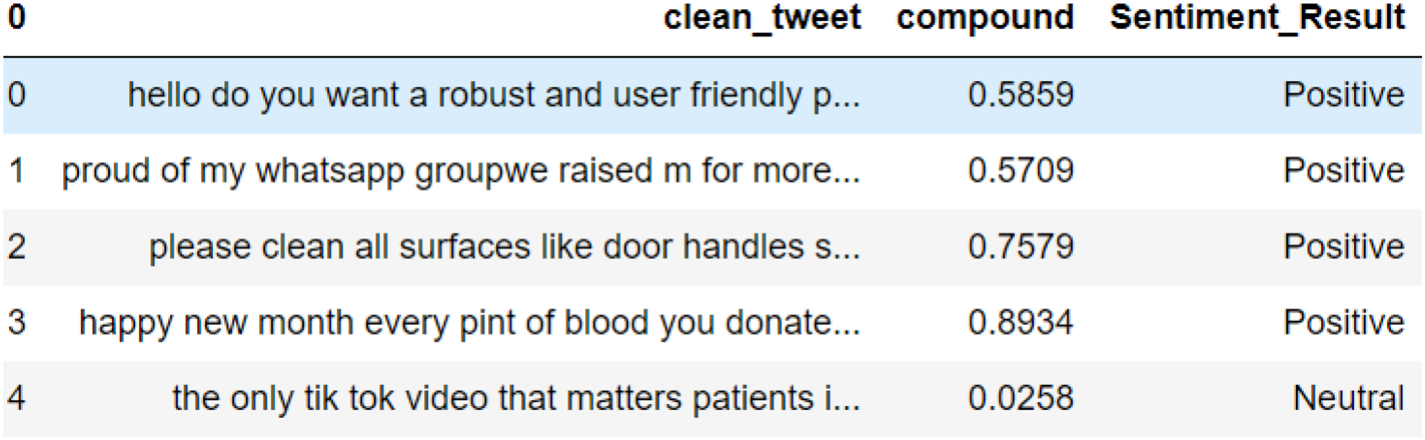
Diagram showing category distribution of sentimental analysis using VADER

As shown in Fig. 6 unlike TextBlob, which employs subjectivity and polarity, this technique divides sentiments into four categories: positive, negative, neutral, and compound.

331,632 instances proved that the authors are positively sentimental, 260,641 instances were negatively sentimental, while 240,388 were neutral in their opinions. The study using VADER analyser as shown in Table 4 shows that authors have high positivity of scaping through the pandemic period.

**Table 4:** Diagram showing the frequency distribution of sentimental analysis using VADER

Sentiment	Count	%
Positive	331,632	39.8
Neutral	260,641	31.3
Negative	240,388	28.9

#### Tweets classification by VADER

**Fig. 7:**
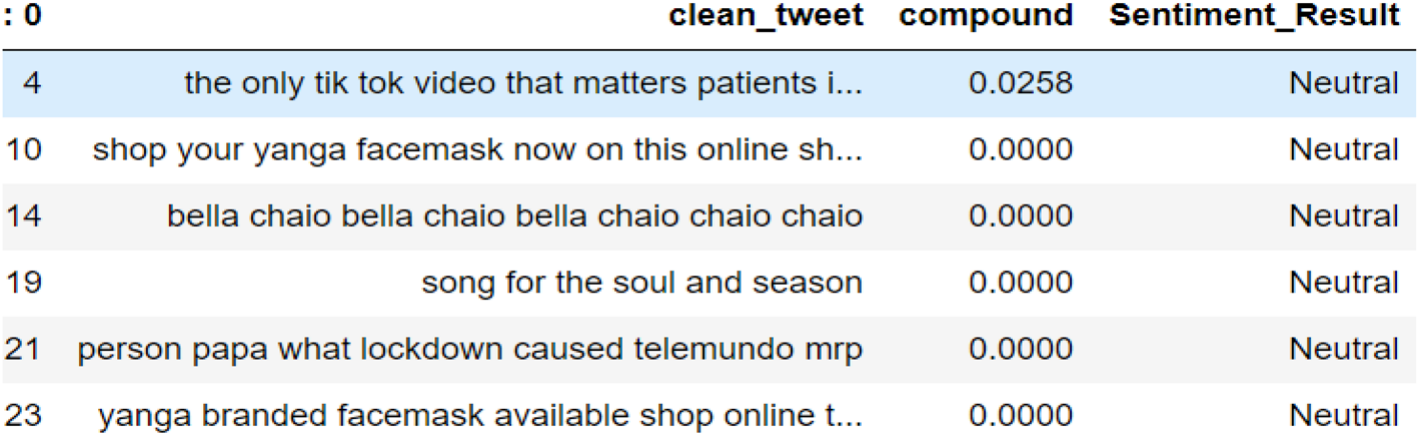
List of tweets classified by VADER as neutral sentiment

**Fig. 8:**
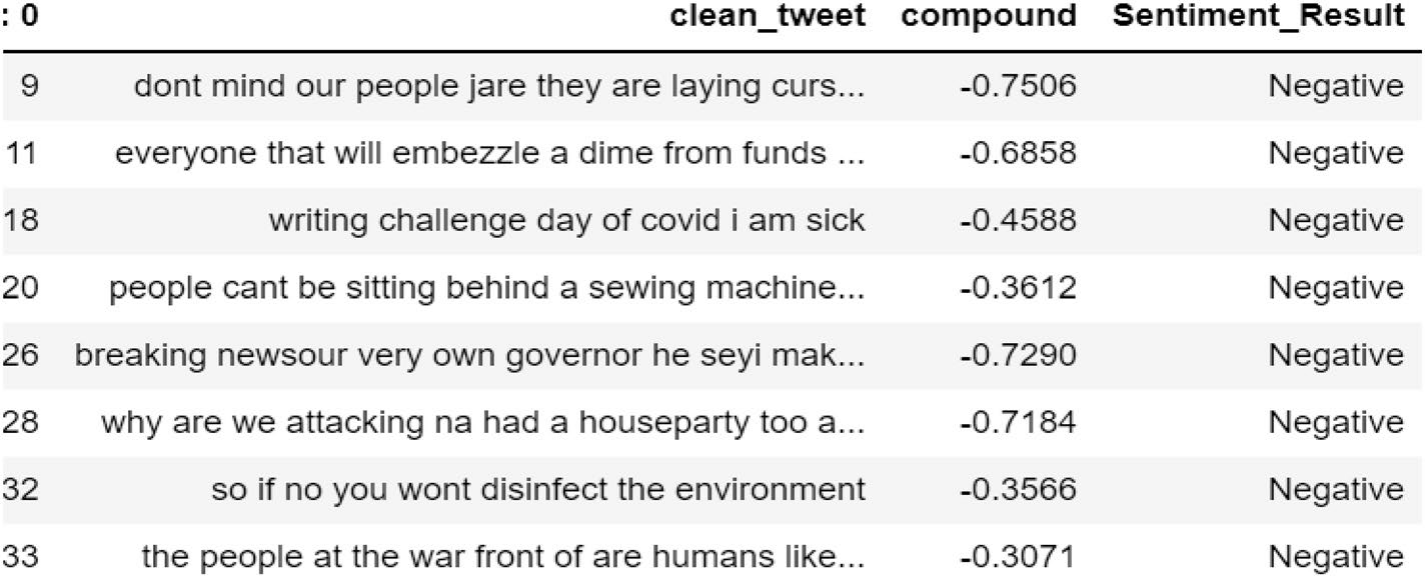
List of tweets classified by VADER as negative sentiment

**Fig. 9:**
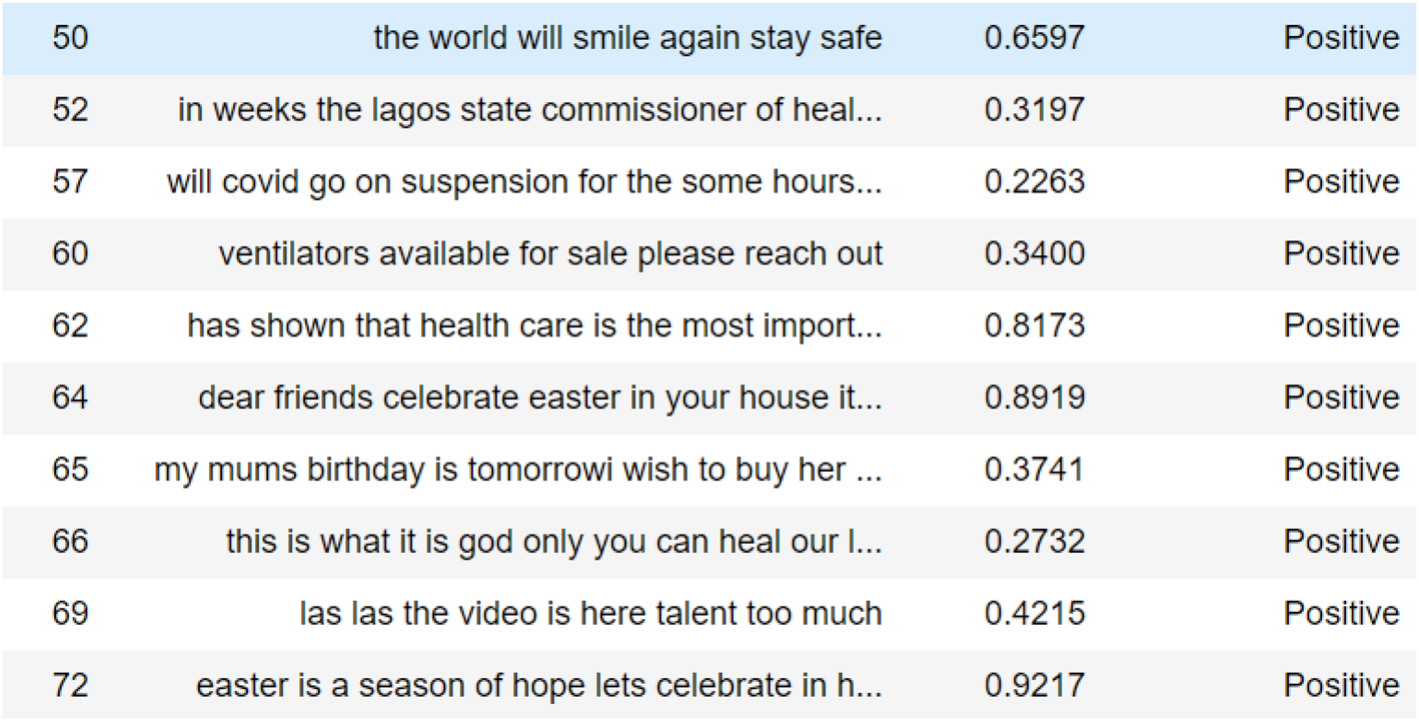
List of tweets classified by VADER as positive sentiment

From the study, as shown in Figs. 7, 8 and 9, the tweets were categorized according to their respective sentimental results which are positive, negative, and neutral. It will be appropriate to visualize these tweets according to their results.

#### Word cloud sentiment of tweets category using VADER analyser

**Fig. 10:**
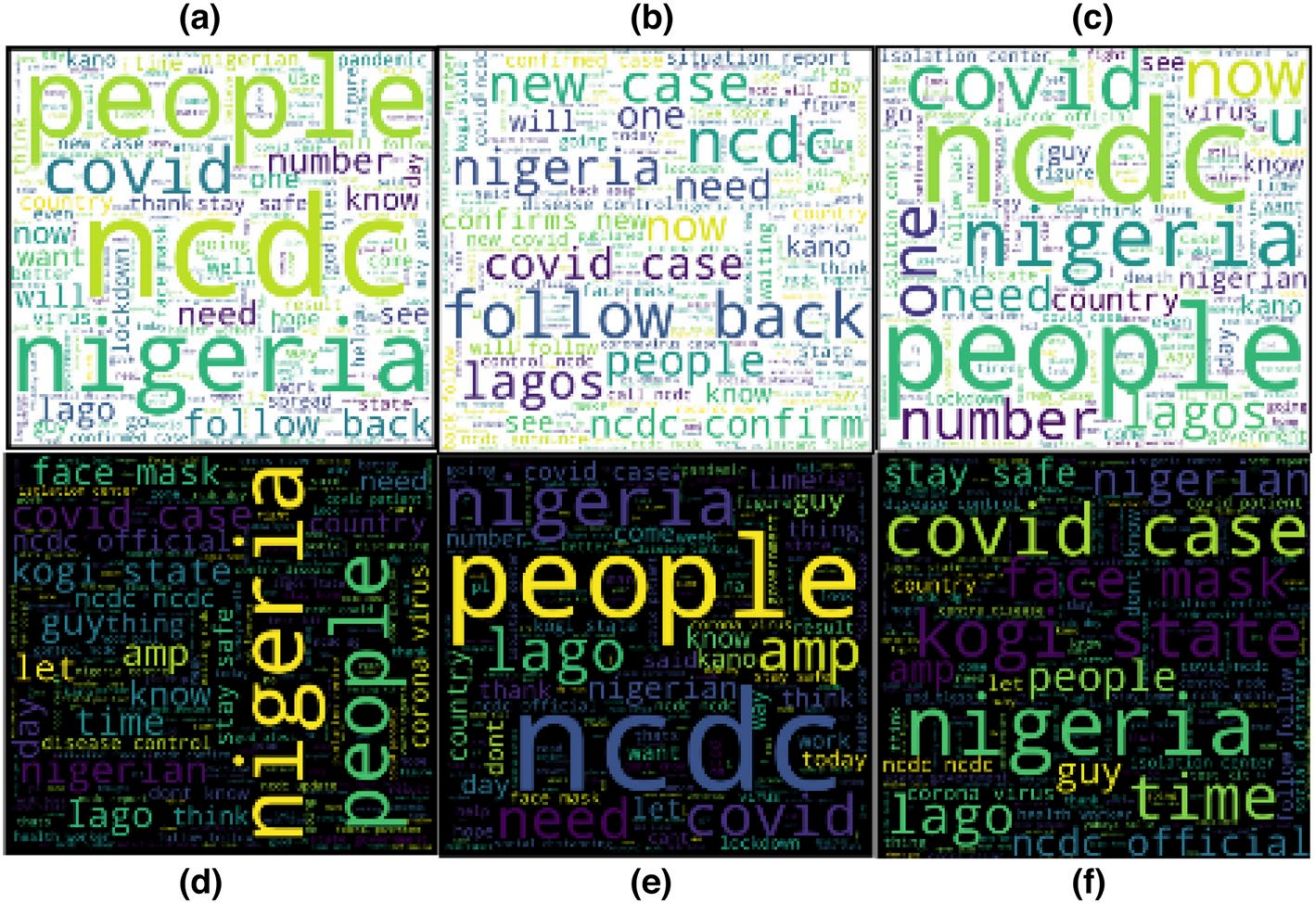
Diagram showing the word cloud of **a** positive tweets using VADER, **b** negative tweets using VADER, **c** neutral tweets using VADER, **d** positive tweets using TextBlob, **e** negative tweets using TextBlob and **f** neutral tweets using TextBlob

As shown in Fig. 10a–c, the study categorizes each sentiment result based on positivity, neutrality, and negativity using the VADER analyser. For proper analysis, a word cloud is used to visualize each category.

#### Word cloud sentiment of Tweets category using TextBlob

Likewise, as shown in Fig. 10d–f, the study categorizes each sentiment result based on positivity, neutral, and negativity using TextBlob.

### Topic modelling

This is used to find abstract subjects in a corpus or data table using word clusters and the frequency of each text. The dashboard indicates the weight of the issue in each document because a text normally comprises multiple subjects of varied proportions.

#### Id2word and corpus

The LDA topic model requires two important inputs: a dictionary (id2word) and a corpus shown as:

$$\left[ {\left[ {\left( {0,{ 1}} \right), \, \left( {{1},{ 1}} \right), \, \left( {{2},{ 1}} \right), \, \left( {{3},{ 1}} \right), \, \left( {{4},{ 1}} \right), \, \left( {{5},{ 1}} \right), \, \left( {{6},{ 1}} \right)} \right]} \right]$$

each word in the document is then assigned a unique id by Gensim. A mapping of (word id, word frequency) is presented in the generated corpus. In the first document, the word id 0 appears only once (0, 1). In the same way, the word id 1 appears once, and so on. This is the input to the LDA model.

$$\left[ {\left[ { \, \left( {^{\prime}{\text{comment}}^{\prime}, \, 1} \right), \, \left( {^{\prime}{\text{drop}}^{\prime}, \, 1} \right), \, \left( {^{\prime}{\text{friendly}}^{\prime}, \, 1} \right), \, \left( {^{\prime}{\text{platform}}^{\prime}, \, 1} \right), \, \left( {^{\prime}{\text{robust}}^{\prime}, \, 1} \right), \, \left( {^{\prime}{\text{user}}^{\prime}, \, 1} \right), \, \left( {^{\prime}{\text{want}}^{\prime}, \, 1} \right)} \right]} \right]$$

#### Building LDA topic model

**Fig. 11:**
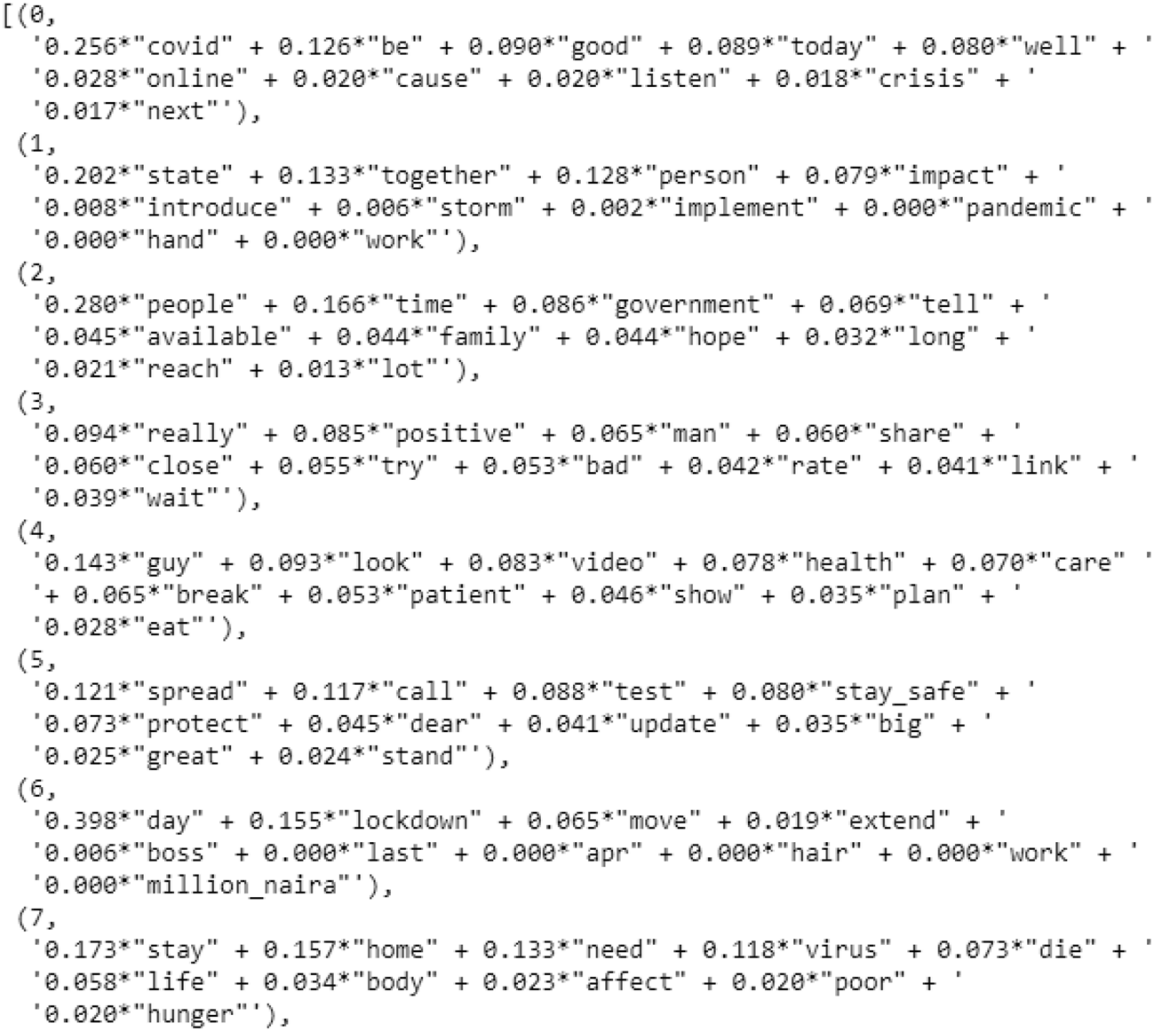
Diagram showing LDA model for 20 topics

As shown in Fig. 11, the LDA model is made up of 20 separate topics, each of which is made up of several keywords, each of which gives a specific amount of weight to the subject. Using lda_model.print_topics(), you can see the keywords for each topic as well as their weight (importance).

Topic 0 is a represented as − 0.256*"covid" + 0.126*"be" + 0.090*"good" + 0.089*"today" + 0.080*"well" + ''0.028*"online" + 0.020*"cause" + 0.020*"listen" + 0.018*"crisis" + ' '0.017*"next".

The top 10 keywords that contribute to this topic are: “covid”', “be”, “good”, “today”, “well”, “online”, “cause”, “listen”, “crisis” and “next” as shown in Fig. 11, and the weight of “covid” on topic 0 is 0.256. The weights represent the importance of a keyword to the topic.

#### Visualize the topic keywords

It is best to inspect the created topics and associated keywords using Intertopic Distance Map after the LDA model has been built via (multidimensional scaling).

**Fig. 12:**
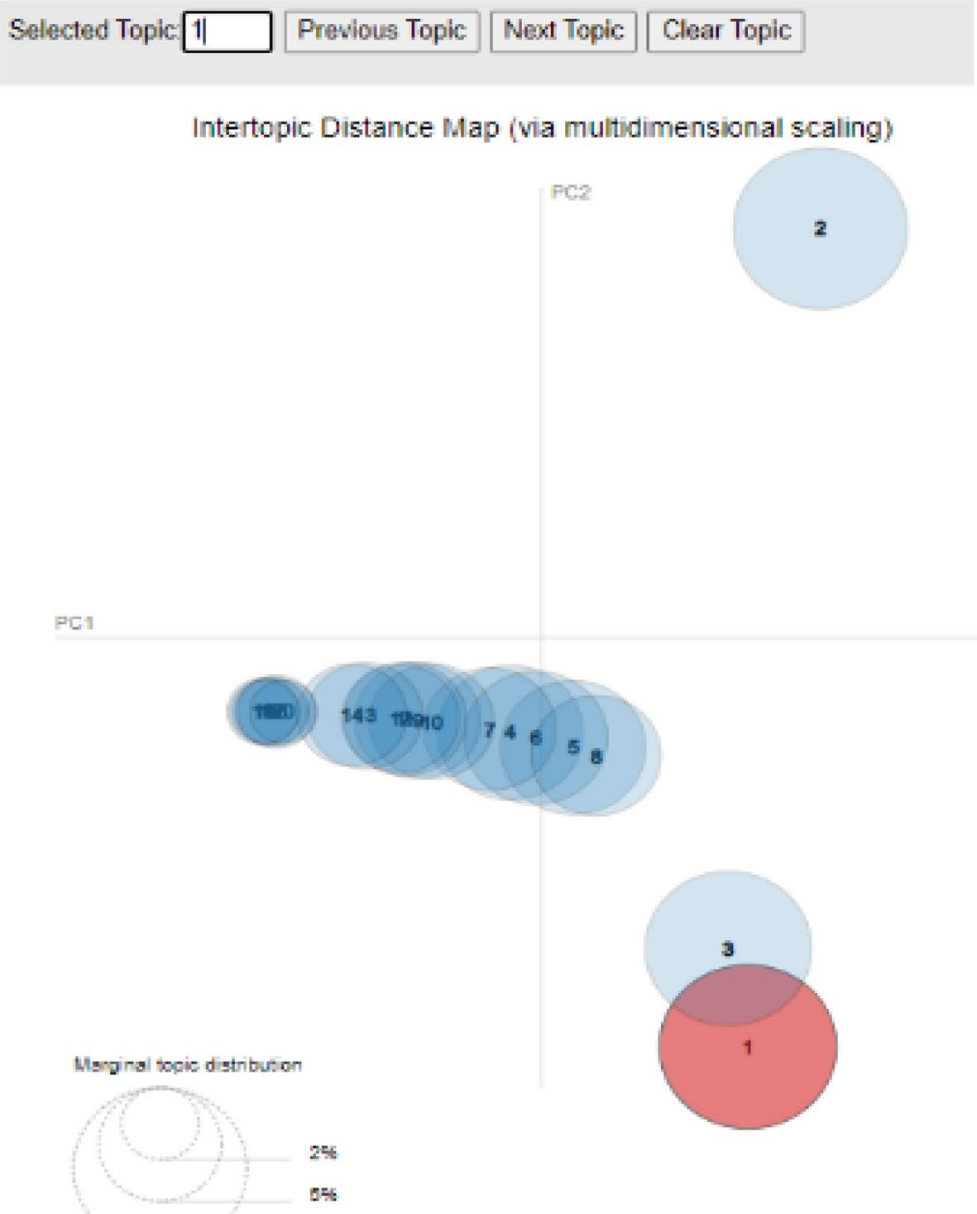
Diagram inspecting topic 1

As shown in Fig. 12, each bubble on the left-hand side plot indicates a topic. The larger the bubble, the more well-known the topic. A strong topic model will have reasonably sized, non-overlapping bubbles spread over the chart instead of being concentrated in one quadrant. Many overlaps, or little bubbles crammed into one area of the graph, indicate a model with too many topics.

**Fig. 13:**
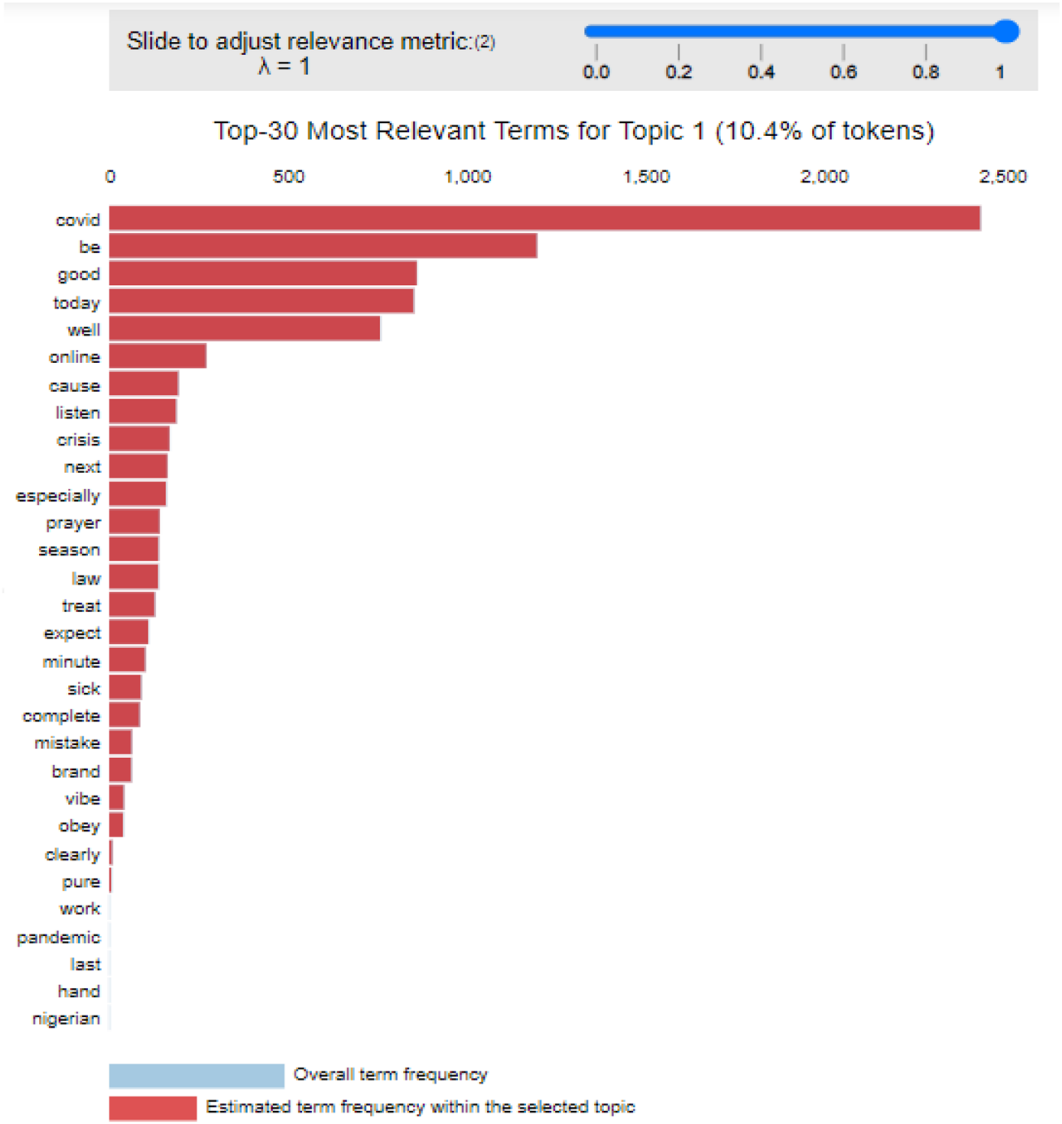
Diagram for text and bar representing each topic selected on the bubbles

As shown in Fig. 13, the text and bars on the right-hand side will update whenever the cursor is moved over one of the bubbles. These are the main keywords that make up the chosen topic such as covid, well, online, pray, sick, and pandemic. This is consistent with the findings in Abdulaziz et al. [7] and Huangfu et al. [24] whose finds also revealed that there were conflicting topics on Twitter throughout the pandemic period.

## Conclusion

In this study, sentiment analysis of Twitter data was conducted using 1,048,575 tweets collected in csv format and converted to a pickle format for easy implementation, these tweets were mined using the COVID-19 hashtags to assess the user’s opinion regarding the current COVID-19 pandemic in Nigeria. From the Twitter data collected, the sentiment analysis of users towards COVID-19 was documented. The study uses TextBlob and VADER sentiment analysers, which resulted in the different analyses as follows: TextBlob sentiment analyser concludes that 46.0%, 36.7%, and 17.3% were neutral, positive, and negative sentiments, respectively, which resulted that the users were mostly neutral about their opinion. VADER analyser returns 39.8%, 31.3%, and 28.9% which implies positive, neutral, and negative sentiments, respectively, which resulted that a larger percentage of the populace being positive about their opinion. The study also concludes by performing a topic modelling analysis of 20 topics to determine the weight of randomly selected corpus by LDA, to know the weight of each token, i.e. how frequent each topic was discussed in the study, then an inter-topic multidimensional scaling was used for the visualizing.

Using content analysis of Twitter data, this study was able to evaluate the performance and assess social media opinion on the trending pandemic concern "Corona Virus". It has demonstrated the extent to which it has sociological, environmental, and economic repercussions in Nigeria, among other things. The study used a sizable sample of users’ thoughts through tweets. To acquire relevant social data from multiple users, semi-structured, self-administered, and Twitter data were used from both verified and unverified accounts. The outcome provides a detailed examination of people's sentiments towards the pandemic in Nigeria.

In Nigeria, a bill against hate speech on social media has recently been introduced by the Senate. If data could be accessed using different API keys from each social media site, a better understanding of how to tackle issues may be gained.

## Data Availability

The dataset is now available at: https://doi.org/10.5281/zenodo.4748715

## References

[1] World Health Organization. (2020) WHO Director-General’s statement on IHR Emergency Committee on novel coronavirus (2019-nCoV). https://www.who.int/director-general/speeches/detail/who-director-general-s-statement-on-ihr-emergency-committee-on-novel-coronavirus-(2019-ncov). Accessed 23 Feb 2021

[2] Manguri KH, Ramadhan RN, Amin PRM (2020). Twitter sentiment analysis on worldwide COVID-19 outbreaks. Kurdistan J Appl Res.

[3] Wang H, Can D, Kazemzadeh A, Bar F, Narayanan S (2012) A system for real-time Twitter sentiment analysis of 2012 U.S. presidential election cycle. In: Proceedings of the ACL 2012 system demonstrations (ACL '12). Association for Computational Linguistics, USA, pp 115–120

[4] Abayomi-Alli A, Abayomi-Alli O, Misra S, Fernandez-Sanz L (2022). Study of the Yahoo-Yahoo Hash-Tag tweets using sentiment analysis and opinion mining algorithms. Inf MDPI.

[5] Marston HR, Shore L, White PJ (2020). How does a (smart) age-friendly ecosystem look in a post-pandemic society?. Int J Environ Res Public Health.

[6] Dubey S, Biswas P, Ghosh R, Chatterjee S, Dubey MJ, Chatterjee S, Lavie CJ (2020). Psychosocial impact of COVID-19. Diabetes Metab Syndr.

[7] Abdulaziz M, Alotaibi A, Alsolamy M, Alabbas A (2021). Topic based sentiment analysis for COVID-19 tweets. Int J Adv Comput Sci Appl.

[8] National Research Council (2013). Public response to alerts and warnings using social media: Report of a workshop on current knowledge and research gaps.

[9] Nichlson (2020) Genomic epidemiology data infrastructure needs for sars-cov-2. Modernizing pandemic response strategies. The National Academies Press, Washington, DC. 10.17226/25879 33030851

[10] Hung M, Lauren E, Hon ES, Birmingham WC, Xu J, Su S, Lipsky MS (2020). Social network analysis of COVID-19 sentiments: application of artificial intelligence. J Med Internet Res.

[11] Gao J, Zheng P, Jia Y, Chen H, Mao Y, Chen S, Wang Y, Fu H, Dai J (2020). Mental health problems and social media exposure during COVID-19 outbreak. PLoS ONE.

[12] Konac A, Barut Y (2021) The role of social media in preventing the COVID-19 pandemic. In: Handbook of research on representing health and medicine in modern media. IGI Global, pp 436–445. 10.4018/978-1-7998-6825-5.ch026

[13] Chakraborty K, Maity P (2020). COVID-19 outbreak: Migration, effects on society, global environment and prevention. Sci Total Environ.

[14] Samuel J, Ali GGN, Rahman MM, Esawi E, Samuel Y (2020). COVID-19 public sentiment insights and machine learning for tweets classification. Information.

[15] Bania RK (2020). COVID-19 public tweets sentiment analysis using TF-IDF and inductive learning models. INFOCOMP J Comput Sci.

[16] Shorten C, Khoshgoftaar TM, Furht B (2021). Deep learning applications for COVID-19. J Big Data.

[17] Alanezi MA, Hewahi NM (2020) Tweets sentiment analysis during COVID-19 pandemic. In: 2020 international conference on data analytics for business and industry: way towards a sustainable economy (ICDABI). IEEE, pp 1–6. 10.1109/icdabi51230.2020.9325679

[18] Ramírez-Sáyago E (2020) Sentiment analysis from twitter data regarding the COVID-19 pandemic. Pre-print. https://www.researchgate.net/publication/346453096

[19] Petersen K, Gerken JM (2021). #COVID-19: an exploratory investigation of hashtag usage on Twitter. Health Policy.

[20] Shi W, Liu D, Yang J, Zhang J, Wen S, Su J (2020). Social bots' sentiment engagement in health emergencies: a topic-based analysis of the COVID-19 pandemic discussions on twitter. Int J Environ Res Public Health.

[21] Dubey AD (2020) Twitter sentiment analysis during COVID-19 outbreak. SSRN 3572023, April 9, 2020. 10.2139/ssrn.3572023

[22] Chakraborty K, Bhatia S, Bhattacharyya S, Platos J, Bag R, Hassanien AE (2020). Sentiment analysis of COVID-19 tweets by deep learning classifiers-a study to show how popularity is affecting accuracy in social media. Appl Soft Comput.

[23] Cao L, Fei-Fei L (2007) Spatially coherent latent topic model for concurrent segmentation and classification of objects and scenes. In: 2007 IEEE 11th international conference on computer/vision. IEEE, pp 1–8. 10.1109/ICCV.2007.4408965

[24] Huangfu L, Mo Y, Zhang P, Zeng DD, He S (2022). COVID-19 vaccine tweets after vaccine rollout: sentiment-based topic modeling. J Med Internet Res.

